# The relationship of *Plasmodium falciparum *humeral immunity with HIV-1 immunosuppression and treatment efficacy in Zambia

**DOI:** 10.1186/1475-2875-8-258

**Published:** 2009-11-18

**Authors:** Jean-Pierre Van Geertruyden, Erika Van Eijk, Francisca Yosaatmadja, Webster Kasongo, Modest Mulenga, Umberto D'Alessandro, Stephen Rogerson

**Affiliations:** 1 Unit International Health, Epidemiology and Social Medicine, Antwerp University, Belgium; 2 Faculty of Earth and Life Sciences, Vrije Universiteit Amsterdam, the Netherlands; 3 Department of Medicine, University of Melbourne, Australia; 4 Tropical Disease Research Center, Ndola, Zambia; 5 Department Parasitology, Institute of Tropical Medicine, Antwerp, Belgium

## Abstract

**Background:**

HIV-1 infection affects malaria humeral immunity during pregnancy, but data for non-pregnant adults are lacking. This study reports the impact of HIV-1 infection and other variables on the level of malaria humeral immunity in adults with clinical malaria and whether humeral immune suppression was a risk factor for treatment failure.

**Methods:**

Sera of 224 HIV-1 infected and 115 uninfected adults were compared for IgG to merozoite antigens AMA-1 and MSP2 (3D7 and FC27 types) determined by ELISA, and for IgG to the Variant Surface Antigens (VSA) of three different parasite line E8B, A4 and HCD6 determined by flow cytometry.

**Results:**

Compared to HIV-1 uninfected adults, AMA-1 IgG was lower in HIV-1 infected (*P *= 0.02) and associated with low CD4 count AMA-1 IgG (*P *= 0.003). Low IgG to all three merozoite antigens was associated with less anemia (*P *= 0.03). High parasite load was associated with low MSP2 IgG 3D7 and FC27 types (*P *= 0.02 and *P *= 0.08). Antibody levels to VSA did not differ between HIV-1 infected and uninfected adults. However, low VSA IgGs were associated with high parasite load (*P *≤ 0.002 for each parasite line) and with treatment failure (*P *≤ 0.04 for each parasite line).

**Conclusion:**

HIV-1 affects humeral responses to AMA-1, but seems to marginally or not affect humeral responses to other merozoite antigens and VSAs. The latter were important for controlling parasite density and predict treatment outcome.

## Background

Humeral responses are of critical importance to blood stage immunity to *Plasmodium falciparum*[1]. Malaria-specific antibodies mediate important anti-parasitic effector functions, including inhibition of cyto-adherence, inhibition of erythrocyte invasion, opsonization for phagocytic clearance, antibody-dependent cyto-toxicity and cellular inhibition[2]. Well-known merozoite antigens are the apical membrane antigen 1 (AMA-1) and merozoite surface proteins (MSPs)[3]. These antigens are involved in erythrocyte invasion[4,5] and are important vaccine candidates[6]. Variant surface antigens (VSAs) are important targets of protective immunity[7], but are also responsible for parasite evasion of the immune system by means of clonal antigenic variation [8-10].

Co-infection with HIV-1 and malaria is common in Africa[11]. HIV-1 infected people with low CD4 count have a higher prevalence of *P. falciparum *malaria infection, disease and treatment failure [12-15]. However, little is known regarding the impact of HIV-1 infection on humeral immunity to *P. falciparum*. Differences in anti-malarial antibody levels have been shown in HIV-1 infected pregnant adults [16-18] and adults hospitalized with AIDS[19], but data for non-pregnant HIV-1 infected individuals are lacking. The impairment of the humeral response to malaria by a HIV-1 infection might partly explain the predisposition of HIV-1 infected adults to malaria disease. Understanding malaria immunity in HIV-1 infected individuals may also have implications for the deployment of future malaria vaccines.

This study assessed the anti-malarial humeral immunity in HIV-1 infected individuals and its association with anti-malarial treatment failure

## Methods

### Patients

This study was conducted in Ndola, Zambia, an area of meso- to hyperendemic malaria[20], between October 2004 and June 2005 within a randomized clinical trial (RCT) comparing the safety and efficacy of artemether-lumefantrine (AL) and sulphadoxine-pyrimethamine (SP) in adults with uncomplicated *P. falciparum *malaria.

The study and the results have been reported elsewhere [20]. Briefly, all individuals aged 15-50 years, attending four peri-urban health centers and with fever (body temperature ≥ 37.5°C), and/or history of fever in the previous 48 hours without any other obvious disease were screened for *P. falciparum *malaria infection and pregnancy (if applicable) and were included if they had a parasite density of at least 1,000 parasites/μL. Additional exclusion criteria were: severe malaria; documented intake of SP or AL two weeks prior recruitment; other cause of fever; evidence of underlying chronic diseases (cardiac, renal, hepatic, malnutrition); pregnancy; history of allergy to study drug or other sulfa drugs; non-resident in the study area.

Clinical history, body temperature and physical findings were recorded. Venous blood (5 ml) was used to prepare blood films, impregnated filter papers (Schleicher & Schuell) for molecular analysis, measurement of hemoglobin (HemoCue^®^) and test for HIV-1 infection, CD4 count and viral load (if HIV-1 infected). Residual plasma was separated, stored and transported at -70°C to be assayed by ELISA and FACS for malarial antibody quantification. Patients were followed up until 45 day post-treatment when CD4 count was reassessed, as a proxy of underlying immune suppression. The study was approved by the ethical and scientific committees of the Institute of Tropical Medicine, Antwerp, Belgium, The Antwerp University, the Tropical Disease Research Centre, Ndola, Zambia and Melbourne Health Research Directorate, Australia.

### Laboratory methods

All laboratory technicians were blinded to clinical data. Thin blood films were fixed with methanol and thin and thick blood films were stained with 10% Giemsa. Parasite densities were determined by microscopy as the number of asexual *P. falciparum *parasites per 200 white blood cells (WBC). Parasitaemia/μl was computed using the actual WBC counts. Internal quality control was organized as recommended by WHO[21]. HIV-1 testing consisted of a screening EIA (Abbott Determine, Abbott laboratories, US) and confirmation of reactive samples by a second EIA (Genie II, Sanofi, Canada). Samples with discordant results were retested with Capillus (Cambridge, Diagnostics, Ireland) whose result was considered as final. CD4 count were performed on all individuals with a direct volumetric absolute CD4 counting instrument (Cyflow^® ^Counter, Partec, Germany)[22]. A FACSCount^® ^instrument (Becton Dickinson, US) was used to validate the Cyflow data and served as a quality control. Based on previous research, a CD4 count of 300/μl at study entry and 450/μL at 45 days follow up was used as a threshold between low and high counts[13]. In order to distinguish recrudescence from reinfection, blood samples, collected on filter paper, were assessed with a nested PCR technique as described previously[20]. HIV-1 RNA was assayed in plasma by the Roche Amplicor HIV-1 Monitor Test, version 1.5 (Roche Diagnostics, Branchburg, NJ, USA).

Several parasite lines (E8B, A4 and HCD6) were used for assessing malarial humeral immunity status. Parasite line E8B, expressing a mix of *var *genes, adheres to CD36 and ICAM-1. Of these *var *genes, A4var (which binds to ICAM-1[23]) is a minor transcript (M Duffy, unpublished). *Var *gene expression of parasite line A4 was maintained by positive selection using monoclonal antibody BC6 [24] and was provided courtesy of Prof C. Newbold. Parasite line HCD6 was derived from a laboratory adapted patient isolate by repeated panning on CD36. HCD6 also expresses a mix of *var *genes and adheres to CD36 but not ICAM-1. Infected erythrocytes (IE) were cultured as described[25]. For flow cytometry, IE with trophozoite-stage parasites were harvested at 7-12% parasitaemia and washed three times in phosphate buffered saline/1% newborn calf serum (PBS+; Commonwealth Serum Laboratories, Melbourne, Australia). IE were resuspended in PBS+ at 10^7^/mL with test serum (1 in 20 dilution, final volume 100 μL) in microtitre plates and incubated at room temperature for 30 min, washed as before, and incubated for 30 min with rabbit anti-human IgG and (after further washing as before) with Alexafluor^® ^488-conjugated donkey anti-rabbit IgG (Invitrogen, Eugene, Oregon USA) and ethidium bromide (final concentration 10 mg/L, PROGEN, Brisbane, Australia). Washed cells were resuspended in 200 μL PBS+ and analyzed on a Becton Dickinson FACSCalibur flow cytometer with Cell Quest software. IE were gated according to ethidium bromide fluorescence, and 1,000 cells positive for ethidium bromide were collected. For each serum sample, the geometric mean Alexafluor 488 fluorescence intensity (MFI) generated by the gated population of IE was recorded. Control serum samples from malaria naïve adults were included in each assay. A positive control pool of hyper-immune serum against VSAs of E8B, A4 and HCD6 was made from the serum of 11 Malawian adults and run in duplicate in each assay. Samples with readings below the mean of the unexposed controls were assigned half the lowest value of that particular VSA variable in order to permit log-transformation; samples given a positive reading were assigned relative values by use of the formula (sample reading minus negative-control reading divided by positive control reading minus negative-control reading) × 100. Samples giving a reading above positive control were further diluted and values were assigned following the same formula and recalculated to the original dilution.

Antibody levels to merozoite antigens were measured by ELISA. Recombinant 3D7 or FC27 MSP2 or AMA-1 proteins (2 μg/ml in PBS, kindly provided by Prof R Anders) were used to coat microtitre plates overnight at 4°C. Plates were blocked for 1 h with PBS/5% skim milk powder (Blotto) and washed five times with phosphate-buffered saline containing 0.05% Tween (PBS/Tween). Samples diluted 1 in 1,000 in Blotto were added to plates (50 μL, in duplicate) and incubated for 2 h. After washing, horseradish-peroxidase-conjugated sheep antibody to human IgG (1 in 2,000; Silenus) was added and the plates were incubated for 2 h then washed three times in PBS/Tween and twice in deionized water. Peroxidase substrate was added and color was developed, plates were read at 415 nm on a Biorad plate reader version 5.2, and OD results were expressed relative to positive and negative controls as for VSA antibodies. Results were also classified as positive (optical density greater than the mean plus two SD of non-exposed sera) or negative. To estimate the "breadth" of immunity a binary variable "low responders" was created. Low responders were defined as having antibody levels below the geometric mean on all three antigens.

Treatment outcomes were defined according to the most recent WHO classification and are described in detailed elsewhere[13,21]. For this report, it was considered that the adequate parasitological and clinical response was the absence of recurrent parasitaemia. Recurrent parasitaemia distinguished new infections from recrudescence by PCR genotyping.

### Statistical analysis

Clinical data were double entered and organized in Epi-info (version 6.04b; Centers for Disease Control and Prevention, Atlanta, GA). All data were exported to STATA version 10.0 (Stata Corporation, College Station, Texas, USA) for analysis. Normally distributed variables were compared by Student's *t *test, non-normally distributed variables by Mann-Whitney's rank sum test (two groups). Differences in proportions were assessed by *χ*^2 ^analysis. Linear regression modeling was used to assess associations between humeral immunity and other factors. For the linear regression modeling, all non-normally distributed variables were log transformed and factors with a *P-*value < 0.10 were retained, as well as factors modifying the regression coefficient of the main determinant by more than 10%. All reported *P*-values are two-sided. Logistic regression modeling was used to assess if humeral immunity was a risk factor for treatment failure. *P*-values < 0.05 were considered significant.

## Results

### Characteristics of the study population

Between October 2004 and June 2005, 339 adult patients were enrolled, 115 (33.9%) of them HIV-1 infected (Table 1). Compared to non-infected, HIV-1 infected patients were older (*P *< 0.001), comprised more women (*P *< 0.001), had lower CD4 count on day 0 (*P *< 0.001) and had lower hemoglobin levels (*P *< 0.001). No differences were found for mean WBC count (*P *= 0.26), parasite load (*P *= 0.92) and body weight (*P *= 0.95).

**Table 1:** Characteristics of study population according to HIV-1 status

Characteristic	No HIV-1 infection (n = 224)	HIV-1 infection (n = 115)	*P *Value
Mean weight, kg (SD)	56.98	56.89	0.95
Number of women, N (%)	98 (43.8)	72 (62.6)	0.001
Mean age, years (SD)	24.7 (9.1)	29.5 (7.6)	<0.001
Mean white blood cell count, *10^9^/L (SD)†	5.3 (1.9)	5.1 (1.7)	0.26
Mean hemoglobin, g/L (SD)	140 (20)	124 (25)	<0.001
Mean Log Viral load HIV-1 RNA copies/μL (SD)		4.87 (0.64)	
Mean (geometric) parasite density/μL (range)†	9,715 (378-153,120)	9,566 (390-158,894)	0.92
Mean (geometric) CD4 count baseline/μL (95%CI)‡	458 (427-492)	259 (242-277)	<0.001
Mean (geometric) CD4 count day45/μL (95%CI) §	839 (769-915)	379 (317-454)	<0.001
Low responders to Combined merozoite antigens, N(%)	42 (18.8)	35 (30.4)	0.02
Low responders to Combined Variant Surface Antigens¶, N (%)	68 (30.4)	33 (28.7)	0.75

*P value based on Students' t- test, Chi square or Wilcoxon Rank -Sum as required

† Data on WBC were missing in 1 patient in the non-HIV-1 infected group and in 2 in the HIV-1 infected group

‡ Data on baseline CD4 count were missing in 6 patients in the non-HIV-1 infected group and in 4 in the HIV-1 infected group

§Data on CD4 count on day 45 used only from patients without parasitaemia; non-HIV-1 infected group n = 108, HIV-1 infected group n = 66

//Low responders are <geometric mean for Merozoïte antigens AMA -1, MSP2 3D7 and MSP2 FC27 and Variant Surface Antigens from parasite lines E8B, A4, and HCD6.

¶After adjustment for CD4 count and age, low responders remained associated with HIV-1 infection (OR:2.40, *P *= 0.01), parasite load (OR: 1.62, *P *= 0.06) and absence of anemia (OR:0.55, *P *= 0.07).

### Antibody levels to merozoite antigens

**Figure 1:**
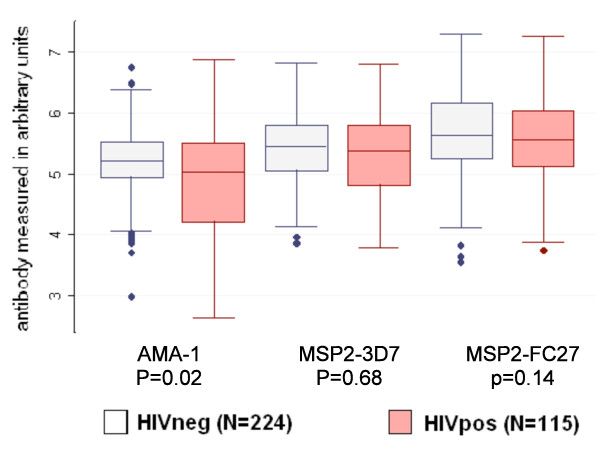
**Anti-Malarial Antibody Levels to Merozoite Blood Stage Antigens by HIV-1 Status**. *P *value by Mann-Whitney's rank sum test.

HIV-1 infected individuals had lower AMA-1 IgG (*P *= 0.02) than HIV-1 negative adults, but this difference in antibody level was not observed for either MSP2 serotypes (*P *= 0.68 and *P *= 0.14) (Figure 1). More HIV-1 infected adults were low responders compared to HIV-1 uninfected adults ((30.4% *vs*18.8%; *P *= 0.02) (Table 1). Overall, low responders (n = 77), compared to high responders (n = 262), were more likely to have a low CD4 count (42.7% *vs *29.9%, *P *= 0.04) and high parasite density (geometric mean 13,461 *vs *8,787 parasites/μL, *P *= 0.01) (Data not shown). After adjustment for CD4 count and age, low responders remained associated with HIV-1 infection (adjusted odds ratio (AOR):2.40; *P *= 0.01), parasite load (AOR:1.62; *P *= 0.06) and had less anemia (AOR:0.55; *P *= 0.07) (Table 1).

**Among HIV-1 infected adults**, low CD4 count at day 0 was associated with low AMA-1 antibody levels (slope:0.9; *P *= 0.003) but not with IgG to the MSP2 serotypes (3D7:slope:-0.04; *P *= 0.92 and FC27:slope:0.21; *P *= 0.38) (Table 2). Parasite load was negatively associated with antibody levels to the MSP2 3D7 (slope:0.25; *P = *0.02) and the MSP2 FC27 serotype (slope:-0.19; *P *= 0.08). No associations between antibody levels with the separate merozoite antigens and CD4 count on day 45 were found in the HIV-1 infected adults. (AMA-1:*P *= 0.69; 3D7:*P *= 0.78; FC27:*P *= 0.76).

**Table 2:** Factors Influencing Antibody Levels to Merozoite Blood Stage Antigen within HIV-1 infected Population (N = 115)

Merozoite Blood Stage Antigen																
**Risk factor**	**AMA -1**				**MSP2 3D7**				**MSP2 FC27**				**Low Responders**			
				
	**Univariate**		**Multivariate**		**Univariate**		**Multivariate**		**Univariate**		**Multivariate**		**Univariate**		**Multivariate**	
								
	**Slope**	***P***	**Slope**	***P***	**Slope**	***P***	**Slope**	***P***	**Slope**	***P***	**Slope**	***P***	**OR**	***P***	**OR**	***P***
																
**CD4 count day 0** **(log^10^/μL)**	**0.68**	**0.03**	**0.9**	**0.003**	0.04	0.86	-0.02	0.92	0.21	0.38	-	-	0.71	0.19	0.44	0.31
**Parasite load** **(log^10^/μL)**	-0.21	0.13	-	-	**-0.22**	**0.03**	**-0.25**	**0.02**	-0.19	0.08	-	-	1.59	0.05	-	-
**White Blood cell Count** (*10^9^/L)	-0.02	0.63	-0.02	0.58	-0.05	0.14	-	-	-0.03	0.64	-	-	1.03	0.21	1.03	0.82
**Hemoglobin day 0** **(g/L)**	**-0.06**	**0.07**	**-0.1**	**0.01**	-0.05	0.03	-	-	-0.03	0.33	-	-	**1.14**	**0.83**	**0.39**	**0.03**
**Viral load** **(log^10 ^RNA copies/μL)†**	-0.11	0.48	-	-	0.02	0.82	-	-	0.1	0.45	-	-	0.95	0.95	-	-
**Age** **(years)**	-0.01	0.53	-	-	0.003	0.74	-	-	0.01	0.43	-	-	1	0.9	-	-
**Gender**	-0.01	0.96	-	-	0.11	0.4	-	-	0.12	0.4	-	-	1.17	0.7	-	-
**Weight** **(Kg)**	-0.003	0.65	-	-	-0.01	0.09	-	-	-0.01	0.02	-	-	1.02	0.12	-	-

Values that reached statistical significance in multivariate analysis are indicated in bold † Viral load was not included in multivariate analyses due to many missing values (35/80)

### Antibody levels to variant surface antigens

**Figure 2:**
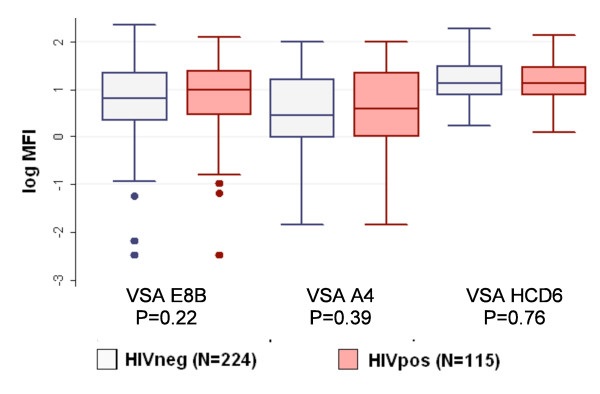
**Total IgG reactivity to Variant Surface Antigens by HIV-1 Status**. *P *value by Mann-Whitney's rank sum test.

HIV-1 infected individuals and uninfected individuals had similar MFI for all three parasite lines expressing different VSAs (E8B:*P *= 0.22; A4:*P *= 0.39; HCD6:*P *= 0.89) (Figure 2). In all patients, regardless of the HIV-1 status, high parasite density (AOR:1.70; *P *= 0.02) and weight (AOR:1.03; *P *= 0.02) were associated with low response to all VSAs.

**Among the HIV-1 infected adults**, parasite density was negatively associated with antibody levels to all VSAs, but an interaction between parasite density and WBC count was found. HIV-1 infected adults with a WBC count ≥ 4,900/μL had a higher parasite load than those <4900/μL (*P *= 0.02). In subjects with a WBC count ≥ 4,900/μL, the parasite density was inversely associated with all VSA antibody levels (E8B:slope:-0.72; *P *= 0.003; A4:-0.72;*P *= 0.002; HCD6:-0.24; *P *= 0.005) (Table 3). Within the HIV-1 infected population, high parasite density (AOR:2.17; *P *= 0.05) and low WBC count (AOR:0.78; *P *= 0.07) were associated with a low response to all three VSAs.

**Table 3:** Factors Influencing Antibody Levels to Variant Surface Antigen within HIV-1 infected Population (N = 115)

Variant Surface Antigen																
**Risk factor**	**VSA E8B**				**VSA A4**				**VSA HCD6**				**Low responders**			
				
	**Univariate**		**Multivariate**		**Univariate**		**Multivariate**		**Univariate**		**Multivariate**		**Univariate**		**Multivariate**	
								
	**Slope**	***P***	**Slope**	***P***	**Slope**	***P***	**Slope**	***P***	**Slope**	***P***	**Slope**	***P***	**OR**	***P***	**OR**	***P***
																
**CD4 count day 0 (log^10^/μL)**	-0.44	0.19	-	-	-0.41	0.23	-	-	-0.05	0.71	-	-	1.66	0.52	-	-
Parasite load (log^10^/μL)*	**-0.29**	**0.05**	**-0.72**	**0.003**	**-0.3**	**0.04**	**-0.72**	**0.002**	**-0.11**	**0.07**	**-0.24**	**0.005**	**1.78**	**0.12**	**2.17**	**0.05**
**White Blood cell Count** (*10^9^/L)	0.07	0.21	-	-	0.04	0.46	-	-	0.03	0.12	-	-	0.83	0.18	0.78	0.07
**Hemoglobin day 0 (g/L)**	0.01	0.83	-	-	-0.02	0.65	-	-	0.001	0.97	-	-	0.81	0.62	-	-
**Viral load** **(log^10 ^RNA copies/μL)†**	0.13	0.49	-		-0.05	0.77	-	-	-0.01	0.92			0.83	0.62	-	-
**Age** **(years)**	-0.01	0.3	-	-	-0.01	0.14	-	-	-0.01	0.14	-	-	1.03	0.34	-	-
**Gender**	0.22	0.22	-	-	0.17	0.36	-	-	0.01	0.92	-	-	0.65	0.32	-	-
**Weight** **(Kg)**	-0.01	0.12	-	-	-0.01	0.05	-	-	-0.01	0.06	0.003	0.19	1.02	0.16	-	-

Values that reached statistical significance in multivariate analysis are indicated in bold

* Stratified on white blood cell count (median 4.9), statistical significance only reached in subgroup with white blood cell count _ 4.9 (median)

† Viral load was not included in multivariate analyses due to many missing values (35/80)

Antibody levels to VSA of E8B and A4 were inversely related to CD4 count at day 45 (both *P *= 0.02) though there was an interaction between initial parasitaemia and CD4 count at day 45 (*P *= 0.05. In HIV-1 infected with a CD4 count at day 45 <450 cells/μL, no association between parasite load and antibody levels to VSA E8B or A4 was found. In HIV-1 infected with a CD4 count ≥ 450 cells/μL on day 45, antibody levels to VSA of E8B and A4 were inversely associated with parasite density (both *P *= 0.03).

### Malaria humeral immunity and malaria treatment failure

Of the evaluable subjects, 82.8% (222/268) patients had an adequate parasitological and clinical response at day 45. PCR genotyping identified 16 patients with new infections (6.0%) and 30 patients with recrudescence (11.2%).

No difference in antibody levels to merozoite antigens or VSA was found between patients successfully treated for malaria and those with recurrent parasitaemia. However, after PCR correction, high antibody levels to the VSAs of E8B, A4, and HCD6 protected for recrudescence *P *= 0.02; *P *= 0.02 and *P *= 0.04 respectively) (Table 4). Additional risk factors for recrudescence were high parasite density (AOR:2.64; *P *= 0.04), anemia (AOR:4.67; *P *= 0.0001) and SP treatment (AOR:7.98; *P *= 0.001). Despite the low power, we observed trends showing SP increasing the risk of new infections (AOR:2.69; *P *= 0.08), and good response to all VSAs decreasing the risk of new infections (AOR:0.16; *P *= 0.08).

**Table 4:** Risk of Recrudescence due to Low Antibody Levels to Blood Stage Malarial Antigens in Total Population (N = 268)

**Parasite Isolate/Antigen**	Odds Ratio*	**95% CI**	**P value**
			
**VSA E8B**	**0.68**	**0.50-0.94**	**0.02**
**VSA A4**	**0.61**	**0.40-0.92**	**0.02**
**VSA HCD6**	**0.28**	**0.08-0.96**	**0.04**
			
**AMA-1**	1	0.58-1.75	0.98
**MSP2 type 3D7**	1.08	0.57-2.07	0.81
**MSP2 type FC27**	1.08	0.57-2.07	0.81

* All results of antibodies to Variant Surface Antigens were adjusted for CD4 count on day 0 in a multivariate logistic regression model. Additional risk factors for recrudescence were high parasite density (AOR:2.64; *P *= 0.04), anemia (AOR:4.67; *P *= 0.0001) and SP treatment (AOR:7.98; *P *= 0.001).

## Discussion

HIV-1 infection increases the risks of malaria infection and disease, and (among immune-compromised patients) decreases responses to anti-malarial therapy [12-14]. Given the central importance of humeral immunity in protecting against malaria, antibody levels to key merozoite antigens and VSA were assessed in adult Zambians with HIV-1 infection and related immune suppression and their association with anti-malarial treatment outcome[13]. Since merozoite antigens and VSAs differ both in their timing of expression and in their conservation or variation between different isolates, they are discussed separately.

### Merozoite antigens: AMA-1, MSP2 3D7 and MSP2 FC27

HIV-1 infection was associated with low antibody levels to AMA-1 and, among HIV-1 infected adults, low AMA-1 antibody levels were associated with low CD4 count. And HIV-1 infected adults were twice as likely as HIV-1 negative adults to have low antibody levels to all three merozoite antigens combined.

Reduced concentrations of antibody levels to AMA-1 have also been demonstrated in HIV-1 infected pregnant Malawian women[18]. In that particular study, antibody levels to AMA1 were not associated with CD4 count, but AMA-1 antibody levels were higher in HIV-1 infected women with malaria infection than those without malaria infection (S Rogerson, unpublished observations).

Acute *P. falciparum *infection induces a short-lived depression in circulating T cell numbers [26,27]. As HIV-1 infection and malaria both influence CD4 count, difficulties arise when assessing associations between those infections and cellular immunity. The positive relationship between the concentration of antibodies to AMA-1 and the CD4 count on enrolment may indicate that low CD4 count directly influences antibody production, as required T cell help is limited. AMA-1 antibody levels at day 0 were not associated with CD4 count at day 45, suggesting that the association between AMA-1 antibody and CD4 count at day 0 may be a function of the degree of T cell reallocation, rather than a direct effect of HIV-1 on antibody levels. Unfortunately, antibody levels were not measured at follow up, when CD4 count was restored, and malaria infection had usually been eliminated: such measurements, and measurement of IgG subclass responses, could provide useful information on the dynamics of immune responses following symptomatic malaria in adults[28]. Limited studies have examined the effect of HIV-1 on antibody immunity to malaria in adults. One such study suggested that HIV-1 infected adults only become vulnerable to malaria when they develop AIDS[19]. Because T and B cell functions are affected early in HIV-1 infection[29], malaria specific humeral immunity might also be affected in the early HIV-1 stages. Antibodies to different targets probably act in an additive or synergistic manner[3] and, under natural exposure, protective immunity to malaria results from high titer antibody levels to multiple antigenic targets [30-32]. To begin to examine the impact of HIV-1 on antibody response to multiple antigenic targets, antibody results for the three merozoite antigens tested were combined [32]. In this study, HIV-1 infected adults were over twice as likely to be "low responders" to this combination of targets, and low responders tended to have a higher parasite load. HIV-1 might decrease antibody to many targets by small amounts; in combination, such defects could predispose to disease or (as these data suggest) to uncontrolled parasite replication.

Somewhat unexpectedly, within the HIV-1 infected population, low responders were less anemic. The recruited patients came from a community where malaria transmission is relatively homogeneous, so differences in exposure and concomitant development of malarial anemia seem unlikely. Second, the major contributor to the hemolytic anemia is the accelerated destruction of uninfected RBC, probably linked to immune mechanisms[33]. Therefore, lower antibody levels might be an independent protective factor for erythrocyte destruction. However, as low responders have higher parasite loads, the overall impact might be limited. Additional research with a longitudinal follow up and a larger panel of antigenic targets is required to determine whether antibody to blood stage parasites influence anemia risk in this already immune-compromised population.

In the whole study population, antibody levels to both MSP2 types were negatively associated with parasite density indicating their direct involvement in parasite control whereas AMA-1 antibody levels were not. The results found for MSP2 IgG confirm studies that have shown associations between antibody levels to blood stage antigens and decreased prospective risk of clinical malaria[6,31,32,34-36].

### Variant surface antigens of the parasite lines E8B, A4, and HCD6

Adult study participants should already have immunity to severe malaria and their antibody repertoire to VSAs may be extensive[3]. It may, therefore, not be surprising that no differences were found in antibody levels to VSA of the different parasite lines between HIV-1 infected and HIV-1 negative adults. Since natural immunity may include the acquisition of a large repertoire of cross-reactive VSA specific antibodies, it may take considerable time until the impact of HIV-1 on humeral responses is apparent. In previous studies, asymptomatic HIV-1 infected individuals maintained antibody levels[37], in contrast to patients with advanced clinical AIDS[19]. Indeed, only increased immune suppression diminishes B cell stimulation, resulting in decreased production of malaria specific antibodies[38]. Nevertheless, reduced antibody production to blood stage antigens and functional abnormalities of VSA specific IgG have been found in HIV-1 pregnant women without clinical AIDS [17,18]. Malaria in pregnancy is characterized by development of immunity to unique VSA expressed by IE capable of sequestering in the placenta [39]. HIV-1 is associated with decreased IgG antibody levels to these VSA, and decreased opsonizing antibody levels that cause phagocytic clearance of such IE [16,18]. Because immunity to pregnancy-associated malaria is only acquired during pregnancy, it may be more susceptible to the effects of concomitant HIV-1, compared to immunity built up by the subjects in this study over a lifetime, much of it preceding HIV-1 infection. The same reasoning might be applicable for children living in malaria endemic areas. Malaria in children is characterized by a development of immunity to VSAs. Low antibody levels to VSAs might be associated with increased clinical malaria incidence and treatment failure and HIV-1 infection might be associated with decreased IgG antibody levels to these VSA. Further research will be needed to assess this hypothesis.

Although VSA antibody levels were not directly associated with HIV-1 infection, they appear to have important roles in controlling malaria parasitaemia in this cohort. Among HIV-1 infected adults with a WBC count ≥ 4,900/μL, antibody levels to VSA were inversely related to parasitaemia. Among all patients, for those with a CD4 count >450/μL on day 45 there was a negative association between parasite load and antibody levels to VSAs of parasite lines E8B and A4. Antibody to VSA operates by preventing sequestration of IE in the vasculature (to avoid splenic clearance), or by opsonization of IE by cytophilic antibodies[40]. Further research should include IgG isotype determination and functional assays of these properties to assess and confirm this finding.

Antibody levels to VSA were similar in patients successfully treated for malaria and the patients who experienced recurrent infection, but high levels of antibody levels to VSAs were associated with decreased risk of recrudescence, suggesting that these antibodies may complement the therapeutic response to the anti-malarial drug[41]. Indeed, an adequate host immunological response is needed in order to maximize the pharmacodynamic properties of an anti-malarial drug[12,41]. As PCR has some technical limitations in distinguishing recrudescences from new infections further research will be needed to conclusively interpret this result[42].

## Conclusion

This study provides evidence that HIV-1 affects humeral responses to AMA-1. Although levels of antibody to the other merozoite antigens and VSA were not or little affected by HIV-1 infection, they appeared to play an important role in controlling parasite density, and the inability to control malaria parasite density is one of the features of HIV-1 immune suppression. Furthermore, recrudescence was associated with low VSA antibody titers to all three parasite lines tested. Specific research in HIV-1 and malaria co-infected adults, with pre-established malaria-immunity, might offer additional insights into malaria immunity.

## Conflict of interests

The authors declare that they have no competing interests.

## Authors' contributions

J-PVG produced the final dataset, contributed to the analysis plan, data interpretation, wrote the paper and takes responsibility for the integrity of the data and the accuracy of the data analysis. EVE did the analysis and wrote the paper. FY performed the ELISA and FACS analysis. WK and MM organized the collection of data, supervised the study trial and contributed to the data interpretation and writing the paper. UD'A contributed to the writing of the paper. SR supervised the laboratory activities related humeral immunity, contributed to the data interpretation and writing of the paper.

## References

[B1] Langhorne J Ndungu FM Sponaas AM Marsh K Immunity to malaria: more questions than answers Nat Immunol 2008 9 725 732 10.1038/ni.f.205 18563083

[B2] Artavanis-Tsakonas K Tongren JE Riley EM The war between the malaria parasite and the immune system: immunity, immunoregulation and immunopathology Clin Exp Immunol 2003 133 145 152 10.1046/j.1365-2249.2003.02174.x 12869017 PMC1808775

[B3] Marsh K Kinyanjui S Immune effector mechanisms in malaria Parasite Immunol 2006 28 51 60 10.1111/j.1365-3024.2006.00808.x 16438676

[B4] Kats LM Cooke BM Coppel RL Black CG Protein trafficking to apical organelles of malaria parasites - building an invasion machine Traffic 2008 9 176 186 18047549 10.1111/j.1600-0854.2007.00681.x

[B5] de Souza W An introduction to the structural organization of parasitic protozoa Curr Pharm Des 2008 14 822 838 10.2174/138161208784041123 18473832

[B6] Nebie I Diarra A Ouedraogo A Soulama I Bougouma EC Tiono AB Chilengi R Theisen M Dodoo D Remarque E Bosomprah S Milligan P Sirima SB Humoral responses to *Plasmodium falciparum *blood-stage antigens and association with incidence of clinical malaria in children living in an area of seasonal malaria transmission in Burkina Faso, West Africa Infect Immun 2008 76 759 766 10.1128/IAI.01147-07 18070896 PMC2223475

[B7] Miller LH Baruch DI Marsh K Doumbo OK The pathogenic basis of malaria Nature 2002 415 673 679 10.1038/415673a 11832955

[B8] Ofori MF Dodoo D Staalsoe T Kurtzhals JA Koram K Theander T Akanmori BD Hviid L Malaria-induced acquisition of antibodies to *Plasmodium falciparum *variant surface antigens Infect Immun 2002 70 2982 2988 10.1128/IAI.70.6.2982-2988.2002 12010988 PMC127986

[B9] Deitsch KW Hviid L Variant surface antigens, virulence genes and the pathogenesis of malaria Trends Parasitol 2004 20 562 566 10.1016/j.pt.2004.09.002 15522665

[B10] Kraemer SM Smith JD A family affair: var genes, PfEMP1 binding, and malaria disease Curr Opin Microbiol 2006 9 374 380 10.1016/j.mib.2006.06.006 16814594

[B11] Van geertruyden JP D'Alessandro U Malaria and HIV: a silent alliance Trends Parasitol 2007 23 465 467 10.1016/j.pt.2007.08.006 17822960

[B12] Hewitt K Steketee R Mwapasa V Whitworth J French N Interactions between HIV and malaria in non-pregnant adults: evidence and implications AIDS 2006 20 1993 2004 10.1097/01.aids.0000247572.95880.92 17053345

[B13] Van geertruyden JP Mulenga M Mwananyanda L Chalwe V Moerman F Chilengi R Kasongo W Van Overmeir C Dujardin JC Colebunders R Kestens L D'Alessandro U HIV-1 immune suppression and antimalarial treatment outcome in Zambian adults with uncomplicated malaria J Infect Dis 2006 194 917 925 10.1086/507310 16960779

[B14] Chalwe V Van geertruyden JP Mukwamataba D Menten J Kamalamba J Mulenga M D'Alessandro U Increased risk for severe malaria in HIV-1-infected adults, Zambia Emerg Infect Dis 2009 15 749 10.3201/eid1505.081009 19402961 PMC2687012

[B15] Mouala C Guiguet M Houze S Damond F Pialoux G Viget N Costagliola D Le Bras J Matheron S FHDH-ANRS CO4 Clinical Epidemiology Group Impact of HIV infection on severity of imported malaria is restricted to patients with CD4 cell counts < 350 cells/microl AIDS 2009 23 1997 2004 10.1097/QAD.0b013e32832f4215 19654499

[B16] Keen J Serghides L Ayi K Patel SN Ayisi J van Eijk A Steketee R Udhayakumar V Kain KC HIV impairs opsonic phagocytic clearance of pregnancy-associated malaria parasites PLoS Med 2007 4 e181 10.1371/journal.pmed.0040181 17535103 PMC1880852

[B17] Dembo EG Mwapasa V Montgomery J Craig AG Porter KA Meshnick SR Molyneux ME Rogerson SJ Impact of human immunodeficiency virus infection in pregnant women on variant-specific immunity to malaria Clin Vaccine Immunol 2008 15 617 621 10.1128/CVI.00378-07 18199738 PMC2292661

[B18] Mount AM Mwapasa V Elliott SR Beeson JG Tadesse E Lema VM Molyneux ME Meshnick SR Rogerson SJ Impairment of humoral immunity to *Plasmodium falciparum *malaria in pregnancy by HIV infection Lancet 2004 363 1860 1867 10.1016/S0140-6736(04)16354-X 15183624

[B19] Migot F Ouedraogo JB Diallo J Zampan H Dubois B Scott-Finnigan T Sanou PT Deloron P Selected *P. falciparum *specific immune responses are maintained in AIDS adults in Burkina Faso Parasite Immunol 1996 18 333 339 10.1046/j.1365-3024.1996.d01-116.x 9229386

[B20] Mulenga M Van geertruyden JP Mwananyanda L Chalwe V Moerman F Chilengi R Van Overmeir C Dujardin JC D'Alessandro U Safety and efficacy of lumefantrine-artemether (Coartem) for the treatment of uncomplicated *Plasmodium falciparum *malaria in Zambian adults Malar J 2006 5 73 10.1186/1475-2875-5-73 16923176 PMC1579224

[B21] World Health Organization Assessment and monitoring of antimalarial drug efficacy for the treatment of uncomplicated falciparum malaria 2003 WHO. Geneva

[B22] Janossy G Jani I Gohde W Affordable CD4(+) T-cell counts on 'single-platform' flow cytometers I. Primary CD4 gating Br J Haematol 2000 111 1198 1208 10.1046/j.1365-2141.2000.02433.x 11167762

[B23] Smith JD Craig AG Kriek N Hudson-Taylor D Kyes S Fagan T Pinches R Baruch DI Newbold CI Miller LH Identification of a *Plasmodium falciparum *intercellular adhesion molecule-1 binding domain: a parasite adhesion trait implicated in cerebral malaria Proc Natl Acad Sci USA 2000 97 1766 1771 10.1073/pnas.040545897 10677532 PMC26510

[B24] Gardner JP Pinches RA Roberts DJ Newbold CI Variant antigens and endothelial receptor adhesion in *Plasmodium falciparum* Proc Natl Acad Sci USA 1996 93 3503 3508 10.1073/pnas.93.8.3503 8622966 PMC39639

[B25] Beeson JG Mann EJ Elliott SR Lema VM Tadesse E Molyneux ME Brown GV Rogerson SJ Antibodies to variant surface antigens of *Plasmodium falciparum*-infected erythrocytes and adhesion inhibitory antibodies are associated with placental malaria and have overlapping and distinct targets J Infect Dis 2004 189 540 551 10.1086/381186 14745713 PMC2613478

[B26] Hviid L Kurtzhals JA Goka BQ Oliver-Commey JO Nkrumah FK Theander TG Rapid reemergence of T cells into peripheral circulation following treatment of severe and uncomplicated *Plasmodium falciparum *malaria Infect Immun 1997 65 4090 4093 9317012 10.1128/iai.65.10.4090-4093.1997 PMC175588

[B27] Van geertruyden JP Mulenga M Kasongo W Polman K Colebunders R Kestens L D'Alessandro U CD4 T-cell count and HIV-1 infection in adults with uncomplicated malaria J Acquir Immune Defic Syndr 2006 43 363 367 10.1097/01.qai.0000243125.98024.da 17079994

[B28] Akpogheneta OJ Duah NO Tetteh KK Dunyo S Lanar DE Pinder M Conway DJ Duration of naturally acquired antibody responses to blood-stage *Plasmodium falciparum *is age dependent and antigen specific Infect Immun 2008 76 1748 1755 10.1128/IAI.01333-07 18212081 PMC2292892

[B29] Miedema F Petit AJ Terpstra FG Schattenkerk JK de Wolf F Al BJ Roos M Lange JM Danner SA Goudsmit J Immunological abnormalities in human immunodeficiency virus (HIV)-infected asymptomatic homosexual men. HIV affects the immune system before CD4+ T helper cell depletion occurs J Clin Invest 1988 82 1908 1914 10.1172/JCI113809 2974045 PMC442771

[B30] John CC Moormann AM Pregibon DC Sumba PO McHugh MM Narum DL Lanar DE Schluchter MD Kazura JW Correlation of high levels of antibodies to multiple pre-erythrocytic *Plasmodium falciparum *antigens and protection from infection Am J Trop Med Hyg 2005 73 222 228 16014863

[B31] Gray JC Corran PH Mangia E Gaunt MW Li Q Tetteh KK Profiling the antibody immune response against blood stage malaria vaccine candidates Clin Chem 2007 53 1244 1253 10.1373/clinchem.2006.081695 17510307

[B32] Osier FH Fegan G Polley SD Murungi L Verra F Tetteh KK Polley SD Conway DJ Holder AA Bacarese-Hamilton T Riley EM Crisanti A Breadth and magnitude of antibody responses to multiple *Plasmodium falciparum *merozoite antigens are associated with protection from clinical malaria Infect Immun 2008 76 2240 2248 10.1128/IAI.01585-07 18316390 PMC2346713

[B33] White NJ Landau N Gautret P Sherman IW Malaria pathophysiology Malaria: parasite biology, pathogenesis and protection. Washington D.C 1998 378 379

[B34] Metzger WG Okenu DM Cavanagh DR Robinson JV Bojang KA Weiss HA McBride JS Greenwood BM Conway DJ Serum IgG3 to the *Plasmodium falciparum *merozoite surface protein 2 is strongly associated with a reduced prospective risk of malaria Parasite Immunol 2003 25 307 312 10.1046/j.1365-3024.2003.00636.x 14507328

[B35] Polley SD Mwangi T Kocken CH Thomas AW Dutta S Lanar DE Remarque E Ross A Williams TN Mwambingu G Lowe B Conway DJ Marsh K Human antibodies to recombinant protein constructs of *Plasmodium falciparum *Apical Membrane Antigen 1 (AMA1) and their associations with protection from malaria Vaccine 2004 23 718 728 10.1016/j.vaccine.2004.05.031 15542195

[B36] Polley SD Conway DJ Cavanagh DR McBride JS Lowe BS Williams TN Mwangi TW Marsh K High levels of serum antibodies to merozoite surface protein 2 of *Plasmodium falciparum *are associated with reduced risk of clinical malaria in coastal Kenya Vaccine 2006 24 4233 4246 10.1016/j.vaccine.2005.06.030 16111789

[B37] Ayisi JG Branch OH Rafi-Janajreh A van Eijk AM ter Kuile FO Rosen DH Kager PA Lanar DE Barbosa A Kaslow D Nahlen BL Lal AA Does infection with Human Immunodeficiency Virus affect the antibody responses to *Plasmodium falciparum *antigenic determinants in asymptomatic pregnant women? J Infect 2003 46 164 172 10.1053/jinf.2002.1088 12643865

[B38] Wabwire-Mangen F Shiff CJ Vlahov D Kline R Serwadda D Sewankambo NK Mugerwa RD Quinn TC Immunological effects of HIV-1 infection on the humoral response to malaria in an African population Am J Trop Med Hyg 1989 41 504 511 2683820 10.4269/ajtmh.1989.41.504

[B39] Rogerson SJ Hviid L Duffy PE Leke RF Taylor DW Malaria in pregnancy: pathogenesis and immunity Lancet Infect Dis 2007 7 105 117 10.1016/S1473-3099(07)70022-1 17251081

[B40] Tebo AE Kremsner PG Luty AJ Fcgamma receptor-mediated phagocytosis of Plasmodium falciparum-infected erythrocytes in vitro Clin Exp Immunol 2002 130 300 306 10.1046/j.1365-2249.2002.01972.x 12390319 PMC1906527

[B41] Mayxay M Chotivanich K Pukrittayakamee S Newton P Looareesuwan S White NJ Contribution of humoral immunity to the therapeutic response in falciparum malaria Am J Trop Med Hyg 2001 65 918 923 11791999 10.4269/ajtmh.2001.65.918

[B42] Juliano JJ Taylor SM Meshnick SR Polymerase chain reaction adjustment in antimalarial trials: molecular malarkey? J Infect Dis 2009 200 5 7 10.1086/599379 19469704 PMC2803033

